# The expression of cyclic adenosine monophosphate responsive element modulator in rat sertoli cells following seminal extract administration

**DOI:** 10.14202/vetworld.2016.1001-1005

**Published:** 2016-09-23

**Authors:** Muslim Akmal, Tongku Nizwan Siregar, Sri Wahyuni, Hamny, Mustafa Kamal Nasution, Wiwik Indriati, Budianto Panjaitan, Dwinna Aliza

**Affiliations:** 1 Laboratory of Histology, Faculty of Veterinary Medicine, Syiah Kuala University, Banda Aceh, Aceh, Indonesia; 2 Laboratory of Reproduction, Faculty of Veterinary Medicine, Syiah Kuala University, Banda Aceh, Aceh, Indonesia; 3 Laboratory of Research, Faculty of Veterinary Medicine, Syiah Kuala University, Banda Aceh, Aceh, Indonesia; 4 Laboratory of Anatomy, Faculty of Veterinary Medicine, Syiah Kuala University, Banda Aceh, Aceh, Indonesia; 5 Department of PGMI, Faculty of Tarbiyah, STAIN Gajah Putih Takengon, Aceh Tengah, Aceh, Indonesia; 6 Student at Veterinary Public Health Graduate Program, Syiah Kuala University, Banda Aceh, Aceh, Indonesia; 7 Laboratory of Clinic, Faculty of Veterinary Medicine, Syiah Kuala University, Banda Aceh, Aceh, Indonesia; 8 Laboratory of Pathology, Faculty of Veterinary Medicine, Syiah Kuala University, Banda Aceh, Aceh, Indonesia

**Keywords:** cyclic adenosine monophosphate responsive element modulator, seminal vesicle extract, Sertoli cells, spermatogenesis

## Abstract

**Aim:**

This study aims to determine the effect of seminal vesicle extract on cyclic adenosine monophosphate responsive element modulator (CREM) expression in rat Sertoli cells.

**Materials and Methods:**

This study examined the expression of CREM on 20 male rats (*Rattus norvegicus*) at 4 months of age, weighing 250-300 g. The rats were divided into four groups: K0, KP1, KP2, and KP3. K0 group was injected with 0.2 ml normal saline; KP1 was injected with 25 mg cloprostenol (Prostavet C, Virbac S. A); KP2 and KP3 were injected with 0.2 and 0.4 ml seminal vesicle extract, respectively. The treatments were conducted 5 times within 12-day interval. At the end of the study, the rats were euthanized by cervical dislocation; then, the testicles were necropsied and processed for histology observation using immunohistochemistry staining.

**Results:**

CREM expression in rat Sertoli cells was not altered by the administration of either 0.2 or 0.4 ml seminal vesicle extract.

**Conclusion:**

The administration of seminal vesicle extract is unable to increase CREM expression in rat Sertoli cells.

## Introduction

The quality of semen containing spermatozoa is highly dependent on its medium fluid called seminal plasma (SP). SP is a part of complex fluid which mediates the function of the ejaculate [1]. Biochemical component of SP is secreted by rete testis, epididymis, and accessory sex glands of male reproduction system [2]. Accessory sex glands consisted of seminal vesicle, prostate, and bulbourethral gland contributed to the volume of the ejaculate. Seminal vesicle secretion is a main component of SP [3].

Seminal vesicle is a hormone-dependent gland producing viscous liquid, yellow fructose, and enriched with seminal fluid (around 60-70% of semen volume) [4] as well as having an important role in male fertility. Seminal vesicle secretes fructose as the energy source for sperm, amino acids, citrate, prostaglandin (PG), and proteins [5]. Secretion of the seminal vesicle can increase the stability of sperm chromatin [6]. Hypofunction of seminal vesicle causes sperm motility disorder and spermatozoa chromatin instability [7]. Furthermore, severe dysfunction of seminal vesicle causes sexual disorder or infertility [5]. Previous research showed that PG contained in the seminal fluid is mostly secreted by seminal vesicle glands [8]. In males, PG is presumed to have a role in increasing libido by triggering the testes to increase the release of steroid hormones [9].

PG stimulates the production of cyclic adenosine monophosphate (cAMP), as a consequence, and induces testosterone synthesis [10]. The addition of PGF2α into semen diluter results in an increase of sperm viability and motility [11].

Testosterone has an important role in maintaining spermatogenesis [12] and male fertility [13].

Sertoli cells transduce signals from testosterone to produce factors needed by the germinal cell for sperm maturation. The bond between testosterone and Sertoli cell will induce two of the molecular pathways, which is the MAP kinase and Ca^2+^ pathways that can induce phosphorylation of cAMP response element binding protein (CREB) [14]. The previous research shows that the administration of seminal vesicle extract increased the quality of spermatozoa, but it was unable to elevate the concentration of testosterone in rats [15].

cAMP response element modulator (CREM), which is highly expressed in spermatid and Sertoli cell [16], regulates gene transcription in response to an increase of cAMP level [17]. CREM was proven to have an essential role in spermatogenesis [18] and spermiogenesis [19]. CREM mutation in mice causes the disturbances to the early stages of spermiogenesis [19], and deletion of CREM gene causes infertility in male mice [20]. Moreover, CREM dysfunction leads to a failure of round spermatid to divide into mature spermatozoa [21]. CREM regulates the expression of some important post-meiosis genes such as protamines and transitional protein genes [22]. Protamines are the major DNA binding proteins in the sperm nucleus that cause DNA condensation and packaging in spermatozoa by histones replacement during spermatogenesis [23]. In addition, protamines are important in maintaining normal sperm morphology, DNA, and motility [24]. Abnormally high or low protamine expression causes DNA fragmentation which consequently leads to lower fertilization rates, poorer embryo quality, and reduced pregnancy rates [25]. This study is subjected to observe the effect of seminal vesicle extract administration on CREM expression in Sertoli cells to the increase of sperm quality.

## Materials and Methods

### Ethical approval

All experimental animals were approved by the Animal Ethics Committee of Faculty of Veterinary Medicine of Syiah Kuala University.

### Seminal vesicle extract preparation

Seminal vesicle was extracted from local Aceh cow testes which were collected from Banda Aceh slaughterhouse. The preparation of 10% seminal vesicle extract was based on the method introduced by Pemayun [26]. A total of 10 seminal vesicle organs were sliced and soaked in methanol for 24 h. The supernatant was obtained and dried using rotary evaporator. Then, 2.5 g of the dried supernatant was added with 10 mg carboxymethylcellulose and diluted into 25 ml normal saline before incubated for 5 min at 37-40°C.

### Treatment

This research used 20 male rats (*Rattus norvegicus*) which were randomly divided into 4 groups. Rats were acclimatized to a new environment for 7 days to avoid stress. During acclimatization, the rats were fed ad libitum, followed by various intraperitoneal injections (26): 0.2 ml of normal saline (K1), 25 μg cloprostenol (K2) (Prostavet C, Virbac S. A), 0.2 ml seminal vesicle extract (K3), and 0.4 ml seminal vesicle extract (K4) [15]. Treatments were administered 5 times at 12-day interval. The rats were euthanized by cervical dislocation and necropsied to collect the testicles for histological observation.

### Histology preparation procedure

Histology slide was prepared according to the method described by Kiernan [27]. Rat testes were immersed in buffered neutral formalin 10% fixative solution, then followed by dehydration process which used increasing concentrations of alcohol (70%, 80%, 90%, 95%, and absolute alcohol, respectively) for 30 min each. The next step was clearing in which the tissue samples were immersed in xylol for 30 min and repeated 3 times. The deparaffinization process was carried out in an incubator at 56-58°C for 30 min and repeated three times. Finally, the samples were embedded in liquid paraffin and let until harden. The samples were sliced into 5 μm using microtome. Every testis was made for four slides and stained by immunohistochemistry staining method.

### Immunohistochemistry staining

Immunohistochemistry staining was done using the avidin-biotin complex method. Initially, the slide was deparaffinized and rehydrated by immersing the slide into xylol solution 3 times for 5 min each, followed by absolute alcohol 3 times for 3 min each, then into a series concentration of alcohol solutions (95%, 90%, 80%, and 70%) for 3 min each, and finally by tap water and distilled water for 10 and 5 min, respectively. Previously, the slides were immersed into 0.3% hydrogen peroxide diluted in methanol for 15 min; antigen retrieval was done by heating the slides in a microwave for 15 min. The slides were then washed using distilled water and PBS pH 7.4 3 times for 10 min. Primary antibody (anti-CREM antibody) was added, and the samples were incubated overnight at 4°C. Then, the slides were left to reach room temperature and washed with PBS 3 times for 10 min each. The secondary antibody (anti-rabbit immunoglobulin G biotin labeled) was added, then the slides were incubated for 30 min at 37°C. Avidin drop was applied, and the slides were incubated in 37°C incubator for 30 min, then washed with PBS 3 times for 5 min each. To visualize the stained samples, the tissues were incubated in 3,3´-diaminobenzidine for 15 min at room temperature, then washed with distilled water for 5 min. Counterstain was done using hematoxylin at room temperature, which then washed by distilled water. Brown coloring on the tissue was the positive reaction toward the immunohistochemistry. The last step of staining was dehydration, clearing, and mounting using Entellan^®^. Observation of the slides was done using a light microscope with 400× magnification.

### Data analysis

The parameter measured in this research was the amount of Sertoli cells expressing CREM. 30 seminiferous tubules in each group were observed under a microscope. Data acquired were analyzed using analysis of variance (ANOVA) then followed by Tukey *post-hoc* test.

### Results

**Figure 1:**
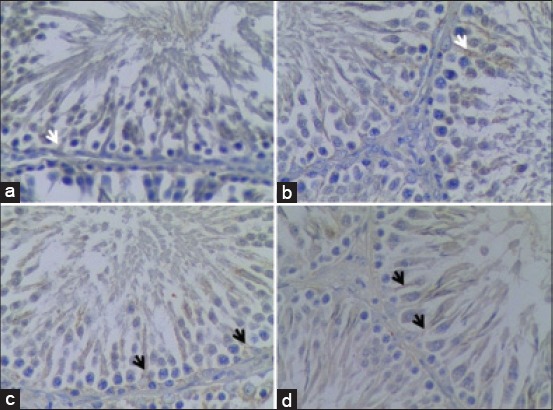
Micrographs of rat testicular tissue (original magnification, ×400). (a) The control group, (b) injected with 25 μg cloprostenol. Both of these tissues revealed no difference in cyclic adenosine monophosphate response element modulator (CREM) expression in Sertoli cells (white arrow). (c and d) injected with 0.2 and 0,4 seminal vesicle extract, respectively – revealed an insignificant difference of the expression of CREM in Sertoli cells. Sections were counterstained with hematoxylin.

Immunohistochemistry staining was used to detect CREM expression in Sertoli cells after the administration of seminal vesicle extract. The statistical values of CREM expression in Sertoli cells are presented in Table-1, whereas the expression of CREM in Sertoli cells can be seen in Figure-1.

**Table-1:** Average CREM expression in Sertoli cells after administration of the seminal vesicle extract for 60 days.

Group	The quantification of Sertoli cells expressing CREM
K1	9.50±0.89^a^
K2	9.80±2.73^a^
K3	11.37±2.88^a^
K4	11.46±2.01^a^

a There was no significant difference between each superscript in the same column (p>0.05). CREM: Cyclic adenosine monophosphate response element modulator

Table-1 showed that CREM expression observed in the rats were injected with 0.2 ml seminal vesicle extract (K3) resulted in a slight increase from 9.50±0.89 to 11.37±2.01 cells compared to the control (K1). The rats which were injected with 0.4 ml seminal vesicle extract (K4) showed a small increase from 9.50±0.89 to 11.46±2.01 compared to the control. Although the results showed an increase in trend corresponds to the increasing doses of seminal vesicle extract (from 0.2 to 0.4 ml), either K3 or K4 showed a significant different with the control (K1) (p>0.05). Furthermore, statistical analysis by ANOVA comparing between control groups (K1 and K2) and treatment groups (K3 and K4) also revealed no significant difference (p>0.05). Thus, the administration of seminal vesicle extract has failed to significantly increase the CREM expression in Sertoli cells.

## Discussion

The previous research showed that seminal vesicle extract is a source of PGF2α. For example, in Bali cattle, PGF2α concentration reached 1750 pg/ml [27]. Administration of seminal vesicle extract containing PGF2α can stimulate testes to increase the release of steroid hormone [9]. PGF2α action stimulates cAMP production which would, in turn, stimulate testosterone synthesis [10]. A signal from cAMP controls KIF17b function modification and directly regulates CREM transcription in male germinal cell post-meiosis [28]. CREM has an important role as spermatogenesis master regulator and is an essential factor during spermiogenesis [29].

Androgens have an important role in spermatogenesis and male infertility [30]. Testosterone androgen is essential for spermatogenesis and secondary sexual characteristic expression [31]. Testosterone is an androgen hormone within testes which is responsible for supporting spermatogenesis. The lack of testosterone will cause infertility in male as a result of spermatogenesis disorder [13]. Sertoli cells are the main cellular target of testosterone signaling which is essential to support the growth and the development of male germinal cells [32]. CREM is not only expressed in spermatid cell but also expressed in testicular Sertoli cells [17].

The bond between testosterone and androgen receptor (AR) in Sertoli cell will induce two testosterone signaling pathways: (1) Testosterone will bind with AR, enabling AR to bind and activate Src tyrosine kinase (SRC), which will stimulate Ras and Raf-1 kinase and activate MAP kinase pathway and (2) testosterone induces Ca^2+^ influx into Sertoli cells that will cause calmodulin (CaM) to stimulate CaM kinase to translocate toward nucleus and transiently phosphorylate CREB in 1 min. The Ca^2+^ also able to stimulate protein kinase C, guanine nucleotide exchange factors, or protein kinase A slowly which would, in turn, stimulate Ras or Ras-like GTP-binding protein which activates MAP kinase pathway. These two pathways could induce phosphorylation of CREB and CREB-mediated gene expression [14].

In addition to its ability to induce CREB phosphorylation, testosterone act in MAP kinase pathway also seems to be able to phosphorylate CREM. Thus, it is reasonable that testosterone is not only able to induce CREB phosphorylation but also able to phosphorylate CREM through MAP kinase pathway. Testicular CREM expression is very important in mouse spermatogenesis [18].

Spermatogenesis is a complex process of producing mature and motile spermatozoa [33]. Failure in CREM expression may cause failure of round spermatid maturation [34]. In addition, CREM is a key factor in the regulation of the expression of post-meiotic genes number during spermatogenesis [18].

However, in this study, the administration of either 0.2 or 0.4 ml seminal vesicle extract failed to increase CREM expression significantly. In agreement with our result, the injection of either 0.2 or 0.4 ml seminal vesicle extract failed to increase the testosterone level although the quality of spermatozoa was significantly elevated after the administration of 0.4 ml of seminal vesicle extract [15]. Therefore, our finding suggested that spermatozoa quality is possibly not entirely regulated by the CREM showing the complexity of molecular signaling mechanism which needs to be studied further. We suppose that the doses of seminal vesicle extract in this research were still low with the result that it was not able to stimulate cAMP production and testosterone synthesis.

## Conclusion

The administration of seminal vesicle extract fails to increase the expression of CREM in Sertoli cells.

## Authors’ Contributions

The manuscript was written by MA and MKN and edited by BP and DA. Planning and execution of this work were under supervision of TNS and MA. This work was carried out by WI, for her Masters degree. Data analysis was done by SW, whereas the expression of CREM by immunohistochemistry technique was assessed under supervision of H. All authors read and approved the final manuscript.

## References

[1] Juyena N. S, Stelletta C (2012). Seminal plasma: An essential attribute to spermatozoa. J. Androl.

[2] Mann T, Lutwak-Mann C (1981). Male Reproductive Function and Semen. Physiology, Biochemistry and Investigative Andrology.

[3] Metafora S, Peluso G, Persico P, Ravagnan G, Esposito C, Porta R (1989). Immunosuppressive and anti-inflammatory properties of a major protein secreted from the epithelium of the rat seminal vesicles. Biochem. Pharmacol.

[4] Lotti F, Corona G, Colpi G. M, Filimberti E, Innocenti S. D, Mancini M, Baldi E, Noci I, Forti G, Maggi M (2012). Seminal vesicles ultrasound features in a cohort of infertility patients. Hum. Reprod.

[5] Noorafshan A, Karbalay-Doust S (2012). Curcumin protects the seminal vesicles from metronidazole-induced reduction of secretion in mice. Acta Med. (Hradec Králové).

[6] Lina S, Hashida N. H, Eliza H (2012). Role of *Habbatus sauda* towards the histological features of nicotine treated male rats seminal vesicle and prostate gland. Biomed. Res., (India).

[7] Gonzales G. F (2001). Function of seminal vesicles and their role on male fertility. Asian J. Androl.

[8] Daniel L. S, Botting R. M, Timothy H. L. A (2004). Cyclooxygenase isozymes: The biology of prostaglandin synthesis and inhibition. Pharmacol. Rev.

[9] Estienne M. J, Harper A. F (2004). Prostaglandins and Boars.

[10] Hess M (2002). The Effects of Prostaglandin F2α, Oxytocin and Gonadotropin Releasing Hormone on Ejaculate Characteristics in The Dog. Thesis. Virginia Polytechnic Institute and State University, Virginia.

[11] Yeste M, Briz M, Pinart E, Sancho V, Garcia-Gil N, Badia E, Bassols A, Pruneda E, Bussalleu E, Casas I, Bonet S (2008). Boar spermatozoa and prostaglandin F2alpha. Quality of boar sperm after the addition of prostaglandin F2alpha to the short-term extender over cooling time. Anim. Reprod. Sci.

[12] Shupe J, Cheng J, Puri P, Kostereva N, Walker W. H (2011). Regulation of Sertoli-germ cell adhesion and sperm release by fsh and nonclassicaltestosterone signaling. Mol. Endocrinol.

[13] Walker W. H (2010). Testosterone signaling and the regulation of spermatogenesis. Spermatogenesis.

[14] Walker W. H, Cheng J (2005). FSH and testosterone signaling in Sertoli cells. Reproduction.

[15] Siregar T. N, Akmal M, Sri W, Hermawaty T, Mulyadi M, Idawati N (2014). The administration of seminal vesicle extract to increase the quality of spermatozoa without affects the spermatozoa and testosterone concentration on white rat (Rattus norvegicus). J. Ked. Hewan.

[16] Wistuba J, Schlatt S, Cantauw C, Von Schönfeldt V, Nieschlag E, Behr R (2002). Transplantation of wild-type spermatogonia leads to complete spermatogenesis in testes of cyclic 3',5'-adenosine monophosphate response element modulator-deficient mice. Biol. Reprod.

[17] Seidl M. D, Nunes F, Fels B, Hildebrandt I, Schmitz W, Schulze-Osthoff K, Müller F. U (2014). A novel intronic promoter of the *Crem* gene induces small ICER (smICER) isoforms. FASEB J.

[18] Javadian F, Estakhr J (2012). Analysis of spermatogenesis and crem gene expression in testis of rat treated with *Matricaria recutita*. Br. J. Pharm. Toxicol.

[19] Nantel F, Monaco L, Foulkes N. S, Masquilier D, LeMeur M, Hendriksen K, Dierich A, Parvinen M, Sassone-Corsi P (1996). Spermiogenesis deficiency and germ-cell apoptosis CREM-mutant mice. Nature.

[20] Blendy J. A, Kaestner K. H, Weinbauer G. F, Nieschlag F, Schütz G (1996). Severe impairment of spermatogenesis in mice lacking the CREM gene. Nature.

[21] Steger K, Klonisch T, Gavenis K, Behr R, Schaller V, Drabent B, Doenecke D, Nieschlag E, Bergmann M, Weinbauer G. F (1999). Round spermatids show normal testis-specific hit but reduced cAMP-responsive element modulator and transition protein 1 expression in men with round spermatid maturation arrest. J. Androl.

[22] Sassone-Corsi P (1998). CREM: A master-switch governing male germ cell differentiation and apoptosis. Semin. Cell Dev. Biol.

[23] Siasi E, Aleyasin A, Mowla J, Sahebkashaf H (2012). Association study of six SNPs in PRM1, PRM2 and TNP2 genes in iranian infertile men with idiopathic azoospermia. Iran. J. Reprod. Med.

[24] Lüke L, Vicens A, Tourmente M, Roldan E. R. S (2014). Evolution of protamine genes and changes in sperm head phenotype in rodents. Biol. Reprod.

[25] Simon L, Castillo J, Oliva R, Lewis S. E. M (2011). Relationships between human sperm protamines, DNA damage and assisted reproduction outcomes. Reprod. Biomed. Online.

[26] Pemayun T. G. O (2007). Kadar prostaglandin F2 alpha pada cairan vesikula seminalis dan produksi sel monolayer vesikula seminalis sapi bali. J. Vet.

[27] Kiernan J. A (1990). Histological & Histochemical Methods: Theory & Practice.

[28] Kotaja N, Macho B, Sassone-Corsi P (2005). Microtubule-independent and protein kinase A-mediated function of kinesin KIF17b controls the intracellular transport of activator of CREM in testis (ACT). J. Biol. Chem.

[29] Nagamori I, Yomgida K, Adams P, Sassone-Corsi P, Nojima H (2006). Transcription factors CREM and Tips40 acts in concert in post-meiotic transcriptional regulation. J. Biol. Chem.

[30] Guido C, Santoro M, De Amicis F, Perrotta I, Panza S, Rago V, Cesario M. G, Lanzino M, Aquila S (2014). Human sperm anatomy and endocrinology in varicocele: Role of androgen receptor. Reproduction.

[31] De Oliveira Souza L. W, Andrade A. F. G, Celeghini E. C. C, Negrão J. A, de Arruda R. P (2011). Correlation between sperm characteristics and testosterone in bovine seminal plasma by direct radioimmunoassay. Rev. Bras. Zootecn.

[32] Griswold M. D (1998). The central role of Sertoli cells in spermatogenesis. Semin. Cell Dev. Biol.

[33] Kanippayoor R. L, Alpern J. H. M, Moehring A. J (2013). Protamines and spermatogenesis in *Drosophila* and *Homo sapiens* A comparative analysis. Spermatogenesis.

[34] Weinbauer G. F, Behr R, Bergmann M, Nieschlag E (1998). Testicular cAMP responsive element modulator (CREM) protein is expressed in round spermatids but is absent or reduced in men with round spermatid maturation arrest. Mol. Hum. Reprod.

